# Dr. Andrews's Cases of Varicose Veins

**Published:** 1807-04

**Authors:** M. W. Andrews

**Affiliations:** Madeira


### Medical and Phyfical Journal.

VOL. XVII.] APRIL, 1807. [NO. 98.

*Printed for R. PHILLIPS, by W. Thorne, Red Lion Court, Fleet Street, London.*

### *To the Editors of the Medical and Phyfical Journal.*

GENTLEMEN,

PERHAPS the following Paper may not convey any thing new to the reader; but, if the cases of such operations that fall to the lot of individuals were never to be made public, the advancement of one most useful branch of the Profession, would be very much retarded; and I am fully convinced, that many prejudices which still exist would be more readily removed by the relation of facts, from which practical inferences may be drawn, than from the most laboured theories.

The operation of tying the vein, in consequence of a varicose state of it, was first practiced in those cases where there was a very irritable ulcer, which was generally met with about the ancle, and which evidently appeared to be kept from healing by that peculiar state of the limb, and was first, I believe, suggested by the advantage that was observed to arise from a bandage applied moderately tight, in such cases as would admit it. It has since been successfully extended by Mr. Home to cases where no ulcer existed, but, I believe, the practice is not very generally followed.

The following cases afford a comparative view of the operation and its consequences under different circumstances, from which, I think, much practical observation may be drawn.

I am, &c.

M. W. ANDREWS.

*Madeira, Sept.* 22, 1806.

### CASE I.

A woman, about 28 years of age, whose occupations required her being almost always in an erect posture, applied to me in January, 1804, in consequence of a varicose state of the veins in her right leg.

About twelve months before, she informed me, she had some complaints requiring the loss of blood, and, as is customary in this country, being an unmarried woman, she was bled in the foot. Shortly after this operation, she experienced great pain in the part where she had been bled, and about three months before she applied to me, perceived the veins at the ancle to be swelled—these gradually became larger, and the varicose state of them extended up the leg and thigh even as high as the groin.

In proportion to their enlargement the pain was also increased; it was at length so great, that she could only bear her leg to be in a horizontal posture, and on no account would it admit of the least pressure, even the sheets touching it often produced an increased uneasiness.

On examining the veins with attention, I found, that the principal trunk which was enlarged, was the Vena Saphena Major; and as it was impossible to apply a bandage sufficiently tight, by which any advantage could be obtained, I determined on including it in a ligature, which I did on the 30th of January, 1804, about mid-way between the knee and groin; the edges of the wound were brought together by adhesive plaster, and lightly dressed.

The pain felt in the course of the veins was of a burning nature, and the superficial veins about the instep were so turgid, as occasionally to give the appearance of blood extravasated under the integuments.

By the 2d of February, the veins were so much reduced in size as not to be perceptible, some of those about the instep excepted. The pain in the groin, which was very great before the operation, had completely subsided; and the ancle, which before would not admit of the smallest pressure, now allowed a flannel bandage to be applied sufficiently tight to give support to the limb.

As the object of the operation appeared to be completely obtained, and as no further good could arise by allowing the ligature to remain until it should be thrown off by suppuration, I removed it by dividing the noose with the point of a pair of dissecting scissars.

The wound, excepting at that point where the ligature had been applied, was completely united, and by the 15th was healed; the veins had no unatural appearance, nor was any pain or disagreeable sensation felt in the leg; and and she was enabled to follow her usual occupation without the smallest inconvenience.

I saw her on the 23d of May following, and learnt that she continued perfectly well.

I have met with many instances of varicose veins on this island; by far the majority have been in females; and on inquiry I have found, they have been bled in the foot at some period before the veins became enlarged; but none attended with sufficient inconvenience to induce them to submit to an operation. Is it not evident, that taking blood from the veins of the foot weaken them so much as to render this complaint more common in those who have undergone that operation?

This prejudice of bleeding in the foot is so great here, that it is sometimes difficult to prevail on an unmarried woman to be bled in the arm, as they consider that operation only proper to be practiced on men, or women during pregnancy.

### Case II.

A Prussian nobleman, of a scrophulous habit of body, and an extremely impatient disposition, had long been subject to severe head-aches and pains in his chest, for the relief of which, about the year 1799, he was recommended to apply a blister on the left leg. This was kept open for ten days. While under the influence of this irritation he imprudently danced. This exertion produced considerable inflammation on the leg, and the consequent pain was very distressing.

From this period he first observed the veins of that leg to become larger than in the other; they gradually increased in size, but were not particularly painful to him.

In 1803 he came to this island, on account of a tendency to pulmonic affections; and as he proposed visiting some of the warmer latitudes before he returned to his own country, he was desirous that something should be done to prevent the further enlargement of these veins, and those consequences which the relaxation of a hot climate might probably occasion; for which purpose he put himself under my care. After having pointed out to him the operation of including the vein in a ligature, as the only thing that I knew which could effectually reduce the varicose state of them, and explaining to him the nature of that operation, I left it for him to determine whether he would have it performed, or submit to the inconvenience arising from the complaint, and risk the consequence of its progress. He decided on the former; and on the 26th of May, 1804, I included the vena saphena major in a ligature, about five inches above the knee, at which part the trunk of the vein was lying very superficial. The wound was dressed as in the former Case.

On the 29th, Tuesday, finding that the object, as far as concerned the immediate effects of the operation, was obtained, I removed the ligature by dividing the noose. The veins were scarcely visible.

Wednesday, 30th. There was a little pain about the wound and neighbouring parts.

Thursday, 31st. A slight degree of inflammation and suppuration, in consequence of which the adhesive plaster was discontinued, and aq. litharg. was applied.

Friday, June 1. Pain the same; suppuration rather increased. Recommended to be perfectly quiet, and to keep the leg in a horizontal posture as much as possible.

Sunday, 3d. Rather more painful, and the inflammation somewhat spread, in consequence of not attending to the directions.

Monday 4th. Went out to an Assembly that was given in honour of the day. Stood much during the evening.

Tuesday, 5th. The consequence of his imprudence, was an appearance of a tumour under the ham, hard and very painful; the inflammation about the wound had spread, and every thing looked very unfavourable. A poultice of bread and milk was applied, and perfect rest, with gentle laxatives, ordered.

Friday, 8th. Nothwithstanding the inconvenience arising from the former party, he could not resist an invitation to dinner; and although he was particularly desired to keep the leg up as much as possible, he not only stood upon it during the day, but walked home to his lodgings in the evening. This, on the best kind of road, would have been extremely improper; but here, where the pavement is so bad, and the situation of his lodgings was on an eminence, is was still more likely to be productive of mischief.

The consequences of this imprudence were, considerable degree of fever, the swelling under the ham increased and extended towards the calf of the leg, and shivering fits, ushering in the formation of matter.

The wound was now fomented and poulticed, with a view to encourage, as much as possible, the discharge from this place. Matter however formed in the leg, and was discharged by an opening near the calf. As there was a coma communication between the abscess that had formed under the ham, and the wound made at the operation, a large portion of the matter was discharged at that opening, by making a considerable pressure with a thick compress under the ham.

Friday 22d. The lower part of the leg was much swelled and inflamed; matter formed, but communicated with the openings already made.

Two or three other abscesses formed, which required to be opened.

Tuesday 26th. The leg was dressed and rolled moderately tight, and a thick compress was applied under the calf, with a view to prevent more sinuses, and to promote absorption, &c. During the whole of this day he felt considerable pain in the leg, more particularly about the calf, and immediately under the ham. The discharge was very copious, passing through the bandage, and was principally blood; the pain suddenly increased towards night, and very much resembled that arising from cramp.

On removing the bandage, a considerable quantity of coagulated blood was discharged from the opening in the leg, and some large sloughs,

Opium not agreeing with his constitution I gave him a large dose of spt. æth. vit. & aq. menth. pip.

Wednesday 27th. Slept but little, and complained of excessive pain in the leg.

On removing the dressings, the discharge was like the preceding evening, and the same kind of discharge continued for five or six days, with large sloughs, some of them nearly three inches in length, but in such a state, that no distinct figure could be traced. The alarm which this created in his mind had a considerable effect upon his general health, as he could in no way reconcile it to himself that the blood was venal, and that it had been for any length of time out of the circulation, but considered it as a recent hæmorrhage.

Some days after the discharge of blood had subsided, the parts not shewing any disposition to heal, a seton was passed from the lower part of the calf to another, about five inches higher up, and another from an opening made under the ham to the wound made at the time of the operation.

In a few days the parts became consolidated, the setons by degrees were removed, the wounds healed, and his general health improved. No appearance of varicose veins could be traced, and when he left this island, and several months after, the state of the leg was perfectly natural.

I am fully aware that the progress of this case is sufficient to make people in general dread the consequences of this operation; and as some respectable practitioners are by no means disposed to recommend it, the publishing this may afford them additional grounds for their objections.

If these consequences were the general attendants on the operation, I should by no means be anxious to dispute the point with them, but I think that a comparative view of both cases is sufficient to support an argument in favour of the operation, as it is very evident that the whole train of unpleasant symptoms in the second case were the immediate consequences of imprudent conduct on the part of the patient; whereas in the former, the patient was completely attentive to every thing that was directed, and no unpleasant consequence ensued.

Is it not probable, that in this case the inflammation had extended to the vein with such violence as to produce a sloughing of its coats, by which the blood instead of being absorbed was discharged through the wound.

This appears to me to have been the case from the following circumstances. The wound at which it was discharged was directly in the course of the vein.

2dly. The blood that was discharged was evidently venal; and,

3dly. From its appearance, it must have been out of the circulation for some time.

### Case of Popltteal Aneurism.

A native of this island had been bathing in the sea, in the month of July, 1805, and while he was swimming, he suddenly felt an acute pain in the ham of the right leg, resembling the sting of a wasp; on examining the part, he did not observe it to be in the least inflamed; he pursued his occupation, which was that of a day-labourer, carrying heavy loads.

About a month afterwards he began to find a continued pain in the knee and under the ham, which was always increased by any particular exertion. At this time a tumour, about the size of a pullet's egg was perceived, and a pulsation distinctly felt in it.

He was admitted into the public hospital in September, and towards the end of that month I saw him, in consultation tation with Doctor Gourlay, and the different medical gentlemen of that establishment.

The swelling was now increased to the size of a large turkey's egg, the pulsation strong, and the pain very great, particularly when pressed.

The leg was somewhat swelled.

The case appeared to me very favourable for operation, and I gave it as my opinion that it ought not to be delayed.

From the operation having never been performed here before, it was proposed by one of the Portugueze practitioners to try the effects of pressure and an astringent application. This plan was continued for four weeks, at the expiration of which time a second consultation was called.

The tumour had now considerably encreased, the pulsation was much stronger, and the whole leg had become much swollen and œdematous.

The man had lost strength, the pain prevented his getting sleep, and the general state of his health was very unfavourable for any operation.

The surgeon, under whose immediate care he was, considered that there was no chance of saving his life but by amputation, not conceiving it possible that a sufficient quantity of blood could be conveyed for the support of the limb by the anastomosing branches, if the artery was tied, and looked upon gangrene of the limb to be the inevitable consequence of such an operation; he therefore declined the operation, as did also the Surgeon, to whose lot the case next fell; and at their request I performed it, on Tuesday, October 29th, in the following manner, assisted by my colleague, Dr. Gourlay.*

Having placed a tourniquet on the thigh, very high up, and laid the limb in a bent position, I made a longitudinal incision, about 3½ inches in length, through the integuments of the thigh, by which the belly of the Sartorius muscle was exposed; and having dissected the integuments so as to raise up the lower edge of the muscle, I brought the blood vessels and nerve into view; then carefully separating the artery from the vein and nerve, I passed a blunt pointed silver needle, armed with a double ligature under the artery, and tied both ligatures, about an inch apart from each other; at the end of each ligature was

* Dr. G. also assisted me in the cases of varicose veins.

was a sharp crooked needle, which was passed through the artery close to each knot, and tied to the opposite end, in the same manner as practised by Mr. Astley Cooper;* after the ligatures were properly secured in this manner, the artery between them was divided by a curved bistory, and the edges of the wound brought together by adhæsive plaster.

On the day of the operation, both legs had the same temperature, viz. 93° of Farenheit's thermometer.

The size of the tumour was taken by passing a piece of paper from the largest part of it, and bringing the two ends to meet on the top of the knee; the paper measured 17 $\frac{7}{8}$ inches.

Wednesday 30th. Some pain in the knee, but not in the tumour; heat in the leg appeared to be greater than in the other; not tried by the thermometer.

Thursday 31st. Swelling of the leg much diminished; pain in the leg and foot considerable, but not in the tumour; heat of operated leg 92°; sound leg 88°: wound dressed; union complete, except where the ligatures were.

Friday, Nov. 1. Pain in the leg very inconsiderable; swelling very much subsided, and no œdema; the heat in each leg taken, was exactly as yesterday; tumour measured as before; paper equal to 17 $\frac{7}{8}$ inches.

Saturday 2d. Leg not painful; heat in each as yesterday.

Sunday 3d. Took bark in substance.

Monday 4th. Heat of operated leg 89°; sound leg 88°.

Tuesday 5th. Tumour measured as before, paper equal to 17 inches.

Sunday 10th. Daily improving in his health; leg, in appearance and heat, perfectly natural; the ligature nearest the knee came away. [12th day.] Tumour much diminished; no pain; can move the knee-joint, which he has long been unable to do.

Monday 11th. The upper ligature came away; can nearly straighten the knee.

Saturday 16th. Eighteenth day from the operation, was able to walk across the ward by the assistance of crutches; tumour diminished since the operation one inch.

Tuesday 19th. Discharged from the hospital.

December 4th. Gradually improving in the use of the knee-

* Vide Medical and Physical Journal, No. xli.

knee-joint: tumour lessened considerably; can walk with one crutch.

March 1806, was now enabled to follow his former mode of life.

### Case III.

A gentleman, 53 years of age, of a delicate constitution, had a paralytic affection in August 1805, which was wholly confined to the right side; of this he soon so far recovered as to be able to walk across the room.

He, however, frequently experienced a great lassitude in the leg on the opposite side; and in the beginning of 1806, observed that the left instep swelled towards evening. In the month of April, the swelling was accompanied with an unusual degree of heaviness and some pain, more especially while in an erect posture.

He now, for the first time, remarked, that the veins of this leg were larger than those of the right, and that they became much fuller after walking, or standing for any time. These circumstances, however, were referred to the extra-exertions of this leg, since his paralytic affection, and appeared to be much influenced by the different degrees of heat to which the limb was exposed.

By June the swelling became more constant, and the heaviness amounted to very severe pain. It was for some time treated as rheumatism. By August the veins had considerably enlarged, and were become completely varicose; he very frequently experienced a severe, acute, but momentary pain, as if a red-hot wire had been passed through them.

His symptoms increased to such a degree as to prevent his taking exercise, and the irritation of this complaint, produced general febrile attacks.

The right ancle began now to swell, and the veins of that leg also were somewhat enlarged and painful.

In October I was consulted, at which period the pain in the leg was so severe that he could not stand for five minutes; the branches of the vena saphena major were very much enlarged; the small superficial veins about the instep were so much distended, as to give the appearance of blood extravasated under the integuments; both ancles were much swelled, and received the impression of the finger when pressed, and he had a considerable degree of fever.

These symptoms were considered to arise from general debility.

I could

I could not, however, agree altogether in that opinion, as it was reasonable to suppose that, whatever impeded the free return of blood from the extremities, would be sufficient to produce, not only all the local inconveniences that he experienced, but such general febrile indisposition as he was then labouring under; and I therefore proposed the operation of including the vein in a ligature, which I did on the 18th of October, 1806.

On the third day from the operation, the ligature was removed, and by the end of the first week the intention of the operation appeared to be completely attained.

From the delicate state of his health, I was particularly anxious that he should be kept very quiet until the wound had completely healed, which required rather more than a month to accomplish. but no untoward circumstance whatever occurred during the cure.

He was ordered bark and port wine, and a generous diet; the veins of the right leg were kept from any further enlargement, by the application of a flannel bandage. He is now able to take exercise without inconvenience.

This case then becomes an additional evidence in favour of the operation; it is a remarkable instance of the advantages to be derived from it, and shews that nothing is to be apprehended when due attention is paid on the part of the patient.

---

### *To the Editors of the Medical and Physical Journal.*

Gentlemen,

